# Brachial pseudoaneurysm of the neonate with partial response to thrombin injections and late spontaneous thrombosis and regression during expectant management

**DOI:** 10.1259/bjrcr.20150383

**Published:** 2016-05-04

**Authors:** Dimitri Parra, Ronald Zuker, Bairbre Connolly

**Affiliations:** ^1^Department of Diagnostic Imaging, Division of Image Guided Therapy, The Hospital for Sick Children, University of Toronto, Toronto, Canada; ^2^Department of Plastic Surgery, The Hospital for Sick Children, University of Toronto, Toronto, Canada

## Abstract

We illustrate the case of a brachial pseudoaneurysm in a 32-week preterm baby male who presented with a large pulsatile mass in the right antecubital fossa, with no clear aetiologic factor. The management of this type of lesion has been controversial and based mainly on case reports. In this case, after obtaining partial response with two thrombin injections, it spontaneously regressed during expectant management. This combination of therapeutic options may be an alternative for the management of complex lesions and, to the best of our knowledge, has not been previously reported.

## Introduction

Pseudoaneurysms (PSAs) develop from a disruption of the arterial wall, which can be secondary to different aetiologies, including inflammation, trauma and iatrogenic causes.^1^ They can have variable presentations, and currently it is accepted that image-guided occlusion methods have replaced surgery as the first choice of treatment.^2^

Brachial artery PSA is a rare condition in neonates.^3^ It has been associated with trauma to the artery during venipuncture.^3,4^ Its management is controversial,^3–5^ with reports of use of surgery and thrombin injection as therapeutic options.

## Case report

**Figure 1.**
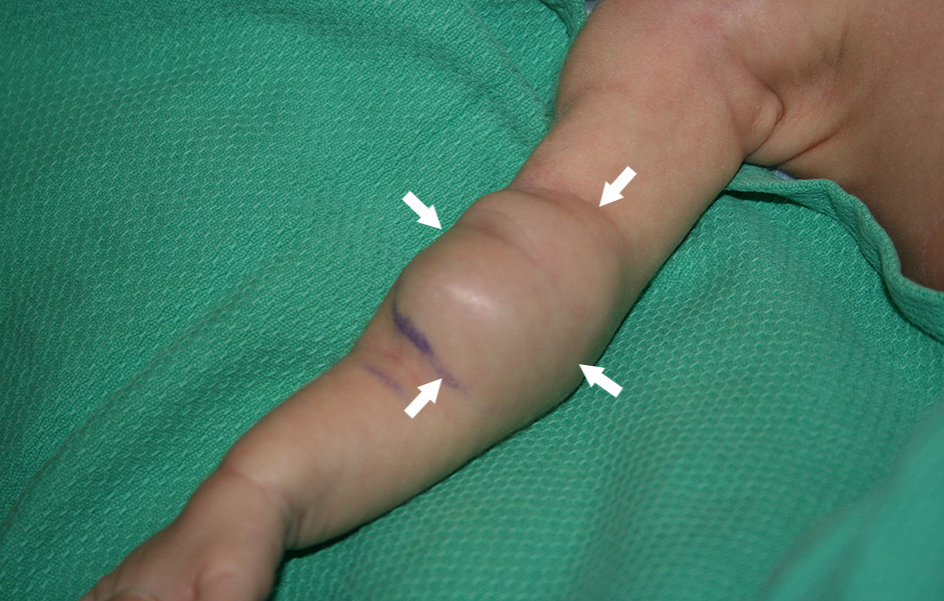
Clinical image of the patient showing a large pulsatile mass in the right antecubital fossa (arrows).

**Figure 2.**
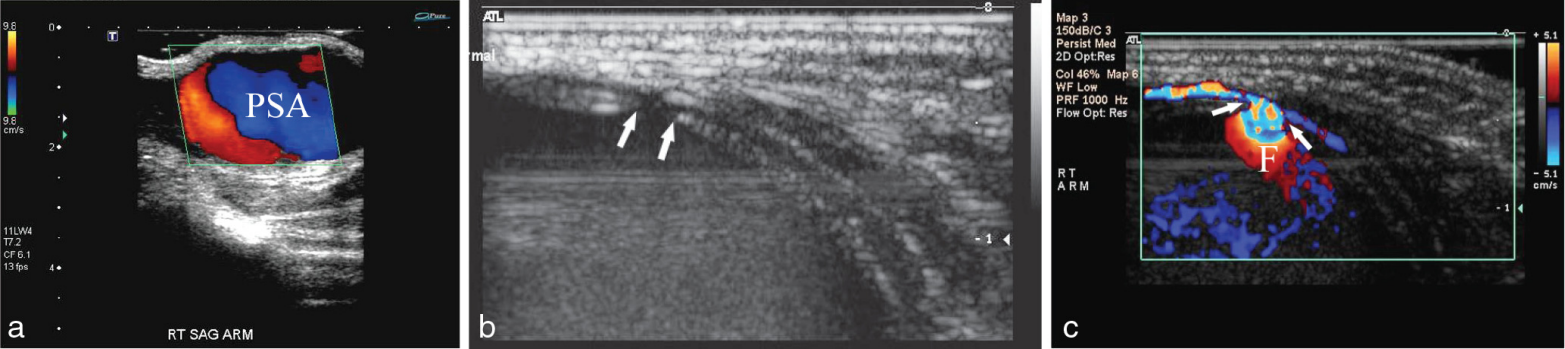
(a) Longitudinal view of the pulsatile mass on colour Doppler ultrasound. This study demonstrated typical features of a large PSA. (b) Transverse view on grey scale ultrasound imaging illustrating that the PSA was secondary to a defect in the posterior wall of the brachial artery (arrows). (c) Colour Doppler study of the same area as in (b) showing that the neck of the PSA was large and wide (arrows), with significant flow (F) into the lesion. PSA, pseudoaneurysm.

**Figure 3.**
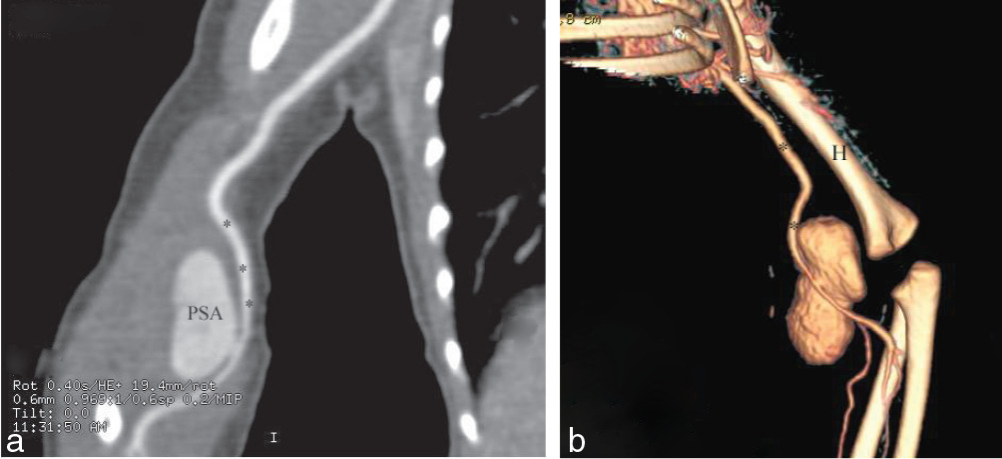
CT angiogram confirmed the sonographic findings. (a) On this longitudinal MIP reformat the PSA is seen displacing the brachial artery (***). (b) Three-dimensional reformat of the lesion performed for surgical planning showing its relationship with the brachial artery (**) and the humerus (H). MIP, maximum intensity projection; PSA, pseudoaneurysm.

A 32-week preterm twin baby male was referred to us at 7 weeks of age (3 kg) with a large, readily palpable pulsatile mass in the upper portion of the right antecubital fossa, which was increasing since day 14 of life (Figure 1). He had been normal at birth, but had had mild respiratory distress for 1 week and prior surgery for hypertrophic pyloric stenosis. Ultrasound examination of the lesion showed a large (3.2 × 2.3 × 1.8 cm) PSA secondary to a small defect in the posterior aspect of the distal brachial artery (visible, wide and short neck) (Figure 2). CT imaging of the area was performed for treatment planning (Figure 3). There was no clear history of trauma, apart from routine venipunctures at another institution. As the baby was very small, the initial consideration was to manage the lesion without surgery until he was a few months old. However, there was considerable clinical and family unease about the progression or catastrophic rupture of the PSA. Given the safety profile of ultrasound compression and thrombin injection in the literature,^1,2^ and after considerable discussion, we decided to intervene initially with ultrasound-guided compression, and if unsuccessful, with percutaneous thrombin injection. Endovascular management was not considered owing to the small size of the parent artery (less than 2 mm) and its critical role in supplying blood to the forearm.

**Figure 4.**
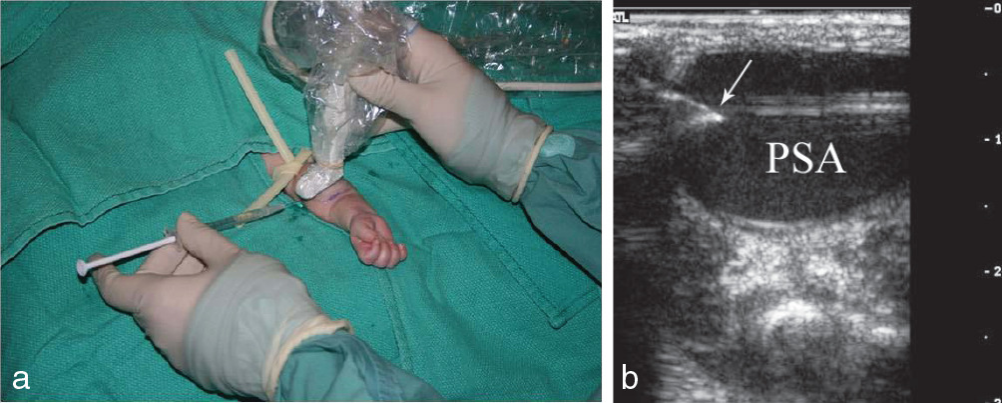
(a) Thrombin was injected into the lesion under real-time sonographic guidance and sterile conditions with a 25-gauge needle. (b) Transverse sonographic view showing the needle (arrow) positioned in the sac of the PSA prior to the injection. PSA, pseudoaneurysm.

**Figure 5.**
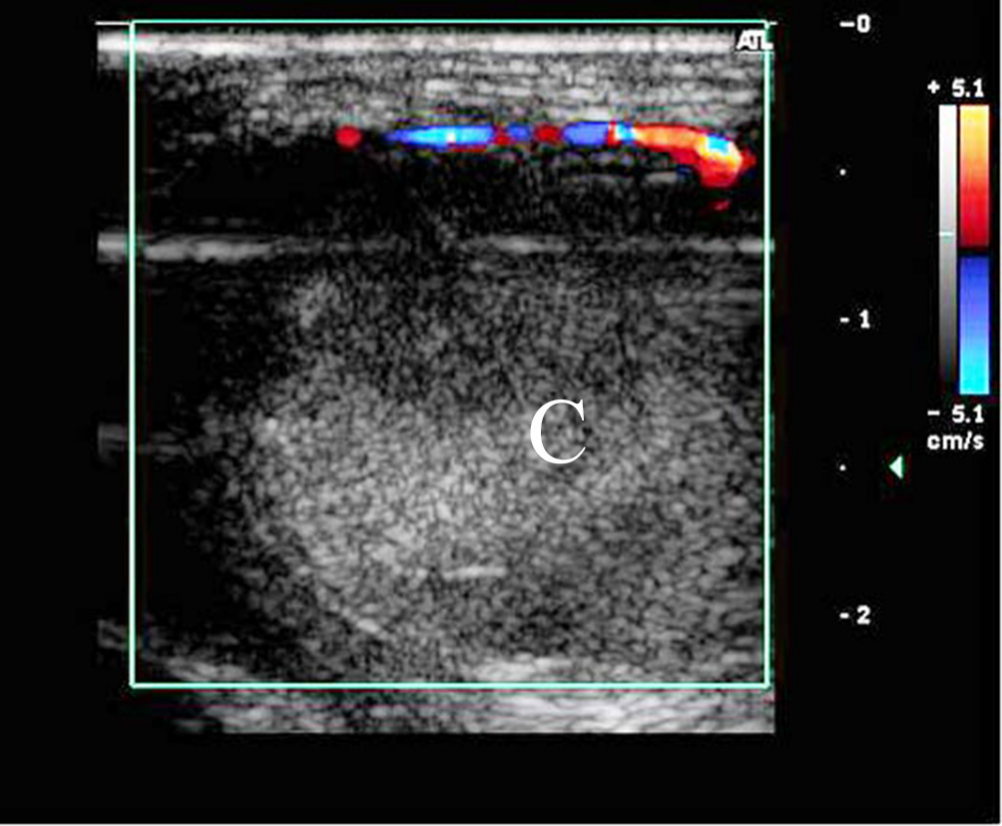
Tranverse sonographic view of the pseudoaneurysm after injecting thrombin (5 units). It shows complete and abrupt cessation of flow in the colour Doppler image within the lesion owing to clot (C) formation.

**Figure 6.**
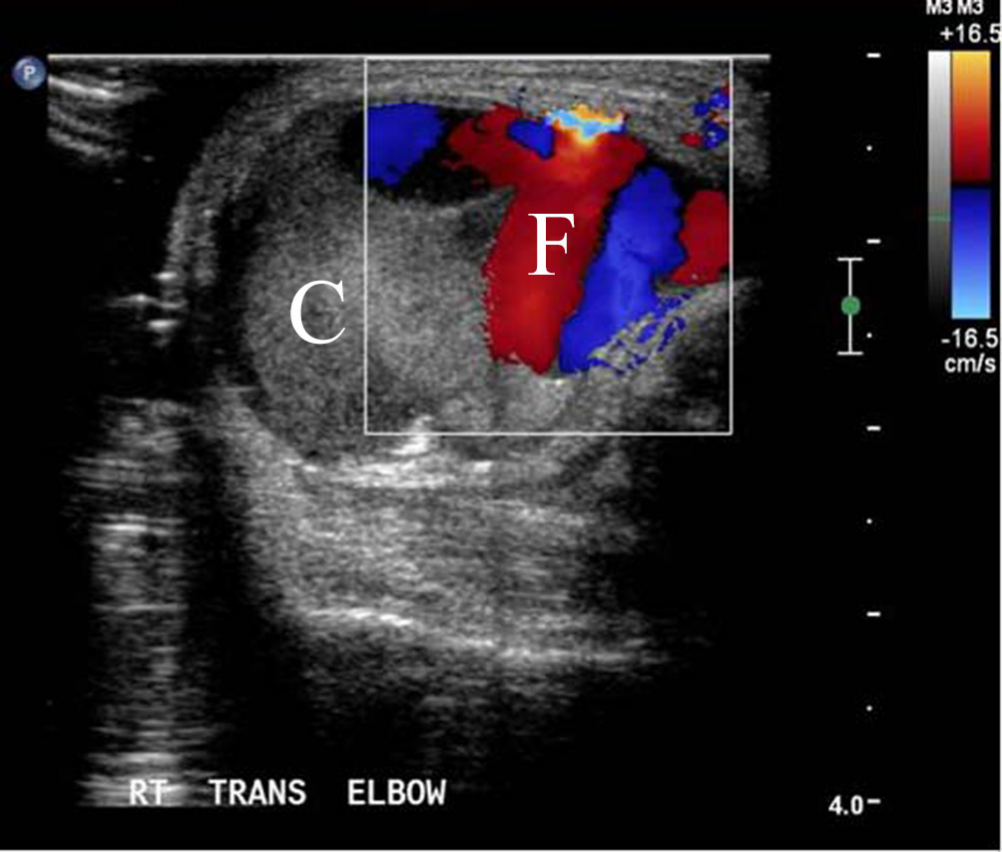
Colour Doppler study performed 24 h after the initial thrombin injection showing partial reopening of the lesion: colour Doppler flow (F) around the clot (C).

**Figure 7.**
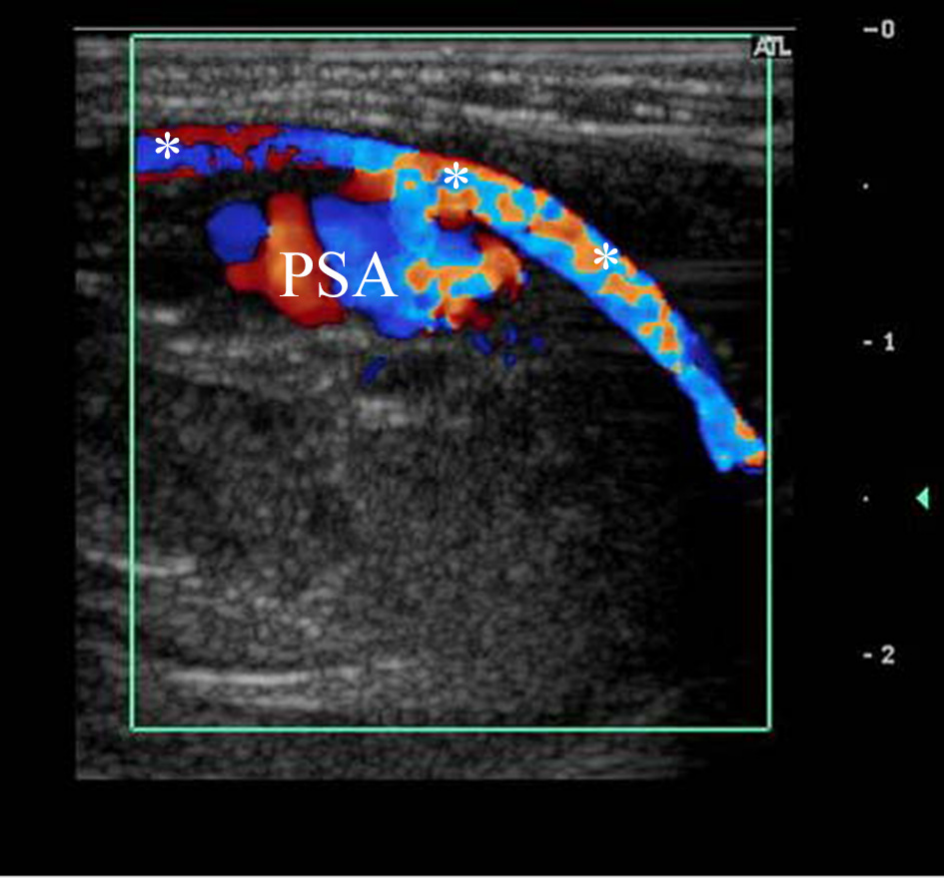
Colour Doppler study in a longitudinal plane after the second thrombin injection. It shows that the PSA reopened again with normal perfusion of the arm, normal flow through the brachial artery (***) and without loss of function. PSA, pseudoaneurysm.

**Figure 8.**
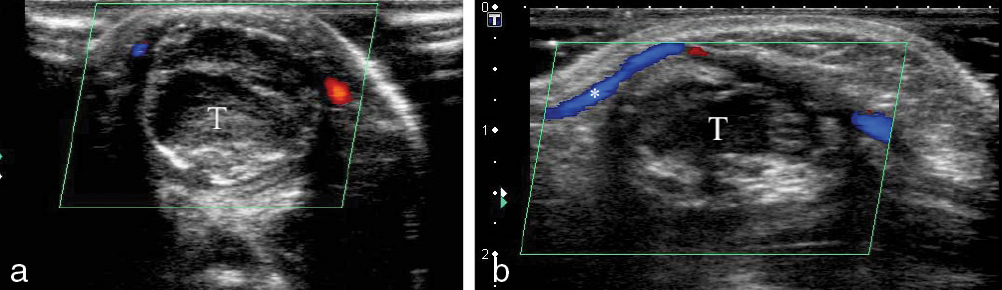
(a) Colour Doppler study on a transverse plane performed after 3 months of expectant management. It shows complete thrombosis (T) and absence of flow within the pseudoaneurysm. (b) Same finding as that in (a) on a longitudinal plane. In this image, good colour Doppler signal can be seen in the brachial artery (*).

Informed consent was obtained and the patient was anaesthetized. Two attempts at ultrasound-guided compression using a 12 Mhz linear probe with a large foot piece placed over the neck of the PSA for 10 min at each attempt were unsuccessful. Under sterile conditions and using sonographic guidance, 5 units of thrombin were injected with a 25-gauge needle into the sac of the PSA, (Figure 4) obtaining complete thrombosis (Figure 5); however, there was immediate compromise of the distal perfusion of the arm. Initial concern was for a thromboembolic event in the arteries of the forearm, although no clot migration was seen on ultrasound during the injection. Later, it became evident that it was owing to the hard mass effect due to the sudden clotting within the sac over the adjacent brachial artery. Intravenous heparin and topical nitroglycerin were used, resulting in total recovery of the extremity; however, the PSA reopened in 24 h (Figure 6). A second injection of 45 units of thrombin was given 7 days later, obtaining good initial results, but partial reopening was observed the following day (Figure 7), mainly in the area adjacent to the neck of the PSA. As there was no functional loss or enlargement of the lesion, conservative expectant management was then adopted. Follow-up with ultrasound imaging showed a persistent decrease in the size of the lesion. Complete thrombosis and absence of flow was observed 3 months after the second injection (Figure 8). The baby’s forearm and hand function remained normal.

## Discussion

PSAs are common vascular abnormalities secondary to a disruption in arterial wall continuity.^1^ They can be secondary to different aetiologies, including iatrogenic causes.^1^ They can present as a pulsatile mass, and management by interventional radiology has replaced surgery as the first line of treatment.^2^

Brachial artery PSA is a rare condition in neonates.^3^ It has been associated with trauma to the artery during venipuctures.^3,4^ Its management is controversial,^3–5^ and based on case reports, surgery is the most commonly used treatment option.^3–4^ The surgical approach requires resection of the lesion with vascular grafting, end-to-end anastomosis or repair of the donor artery,^4^ and is associated with a very good success rate, which has improved with the continuous advancements in microsurgical techniques.

Ultrasound-guided thrombin injection for the treatment of superficial PSA had been widely used with a success rate that ranges from 91% to 100%.^6^ Pezzullo and Wallach^5^ successfully treated a brachial PSA in a 3-week-old patient with 300 units of thrombin.

In our case, surgery was not initially considered owing to the age and weight of the patient. Technically, it did not appear possible to use an endovascular approach owing to the size of the parent artery. Therefore, our option was initially to attempt ultrasound-guided thrombin injection. While performing the first injection, we noted quick thrombosis with significant hardening of the lesion. We believe that this hardening increased the pressure in the compartment of the arm, with secondary compression of the brachial artery and decreased flow in the distal arm. This necessitated heparinization and use of topical nitroglycerin. With the second injection, we only obtained a partial response, probably secondary to the wide and short neck and the associated high intralesional blood flow. Expectant management was undertaken, waiting for the patient to gain weight for a surgical repair. However, the lesion resolved without any further interventions. It is matter of speculation whether or not the occlusion may have been assisted by the partial intralesional thrombosis obtained with the thrombin injections. Expectant management has been mentioned as a therapeutic option; however, it is rarely recommended.^2^

In conclusion, this case illustrates the use of a combination of ultrasound-guided thrombin injection and expectant management in managing a technically challenging PSA from interventional radiology (neck characteristics and size of the parent artery) and surgical (weight of the patient, size and location of the lesion) perspectives.

## Learning points

1. Brachial PSA in neonates is an uncommon condition, which has been associated with trauma to the artery during venipuncture.
2. Its management can be challenging, especially if the PSA neck is wide.
3. As treatment may risk the arterial supply to the arm, a multidisciplinary approach, on a case-to-case basis, is recommended.

## Consent

Informed consent was obtained from the parents of the patient for publication of this case report and unidentified pictures. This was recorded in the patient’s chart as per our hospital policy.
